# Biomonitoring of Occupational Exposure to Mycotoxins Among Swine Farm Workers: An Italian Pilot Study

**DOI:** 10.3390/toxics14070562

**Published:** 2026-06-27

**Authors:** Enrico Paci, Alessandra Chiominto, Anna Rita Proietto, Daniela Visaggio, Paolo Visca, Angela Gioffrè, Raffaella Aiello, Concettina Fenga, Daniela Pigini, Emilia Paba

**Affiliations:** 1 Department of Occupational and Environmental Medicine, Epidemiology and Hygiene, Italian Workers’ Compensation Authority (INAIL), Monte Porzio Catone, 00078 Rome, Italy; a.chiominto@inail.it (A.C.); a.proietto@inail.it (A.R.P.); d.pigini@inail.it (D.P.); e.paba@inail.it (E.P.); 2 Department of Science, Roma Tre University, 00154 Rome, Italy; daniela.visaggio@uniroma3.it (D.V.); paolo.visca@uniroma3.it (P.V.); 3 Department of Occupational and Environmental Medicine, Epidemiology and Hygiene, Italian Workers’ Compensation Authority (INAIL), 88046 Lamezia Terme, Italy; an.gioffre@inail.it; 4 Experimental Zooprophylactic Institute of Southern Italy, Catanzaro Section, 88100 Catanzaro, Italy; aiello.raffaella@outlook.it; 5 Department of Biomedical Sciences, Dental, Morphological and Functional Investigations, University of Messina, 98122 Messina, Italy; concettina.fenga@unime.it

**Keywords:** mycotoxins, occupational exposure, swine farming, biomarkers, oxidative stress

## Abstract

The risk of exposure to mycotoxins in livestock farming is still poorly characterized, particularly in Italy where human biomonitoring data are scarce. Livestock farms represent a high-risk setting due to frequent handling of contaminated feed and dust-generating activities. This pilot study applied a human biomonitoring approach to assess internal exposure to multiple mycotoxins among pig farmers in Southern Italy. Urinary biomarkers of aflatoxin B1 (AFB1), aflatoxin M1 (AFM1), ochratoxin A (OTA), and fumonisin B1 (FB1), together with oxidative stress biomarkers (8-oxo-7,8-dihydroguanine (8-oxoGua), 8-oxo-7,8-dihydro-2′-deoxyguanosine (8-oxodGuo), 8-oxo-7,8-dihydroguanosine (8-oxoGuo), 3-nitrotyrosine (3-NO_2_Tyr), and 5-methylcytidine (5-MeCyt)), were measured in urine samples from 35 workers and 30 non-exposed controls. A sensitive and validated HPLC–MS/MS multi-mycotoxin method was developed and applied. Biomonitoring results were also discussed in relation to previous environmental monitoring. AFM1 emerged as the most frequently detected biomarker in the exposed group, with concentrations above the limit of detection (LOD) in 22.8% of samples; 11.4% exceeded the limit of quantification (LOQ). In contrast, only 10% of the control samples had values above the LOD and none exceeded the LOQ, suggesting a possible contribution linked to occupational tasks. This study provides original biomonitoring evidence of low-dose, mixed mycotoxin exposure among Italian swine farmers and highlights the value of integrating environmental and biological monitoring to improve occupational exposure assessment in livestock production systems.

## 1. Introduction

Mycotoxins are toxic, stable secondary metabolites produced by certain species of fungi (mainly *Aspergillus*, *Fusarium* and *Penicillium*) that contaminate crops, food and feed worldwide [1,2]. These metabolites have been associated with a wide range of adverse health effects, including respiratory irritation, immunotoxicity, hepatotoxicity, and carcinogenicity, depending on the type of toxin, as well as the route and duration of exposure [3,4,5].

Although dietary intake is considered the main source of human exposure to mycotoxins, increasing evidence indicates that occupational exposure—particularly through inhalation of contaminated dust—may represent an additional and relevant contributor to internal dose. In particular, the livestock sector has been identified as a high-risk environmental setting due to frequent handling of cereal-based feed, bedding materials, close contact with animals, and daily tasks that generate large amounts of dust. Therefore, farm workers may be exposed to mycotoxins through multiple pathways, including accidental ingestion, dermal contact, and, importantly, inhalation of contaminated bioaerosols generated during routine activities such as feed handling, animal care, and cleaning operations [6,7,8].

In complex occupational settings such as livestock farms, human biomonitoring (HBM) has emerged as a robust approach to assess overall mycotoxin exposure, as it integrates multiple exposure routes and allows the simultaneous determination of several mycotoxins and their metabolites, capturing not only dietary intake but also inhalation-related occupational exposure [9,10,11].

The use of biomarkers has become increasingly widespread, and research aims to identify new and more specific indicators, as biomarkers have proven to be an effective tool for assessing exposure to xenobiotics. However, some challenges have to be addressed, such as the deep knowledge about the toxicokinetics and the possible metabolites for all relevant mycotoxins. One of the main challenges is the ongoing identification of new metabolites for each mycotoxin and the assessment of their suitability for biomonitoring studies, taking into account both analytical feasibility and the actual representativeness of exposure to the mycotoxin of interest [7,9,11,12].

Available biomonitoring studies in occupational settings remain limited; furthermore, existing evidence indicates that workers are rarely exposed to a single mycotoxin, but rather to complex mixtures, raising concerns about potential additive or synergistic effects that are not adequately addressed by current single-compound risk assessment approaches [7,8,13].

Among livestock systems, swine production has received specific attention at the European level. A landmark study conducted in Portuguese pig farms highlights the presence of several mycotoxin biomarkers in workers’ urine, including deoxynivalenol (DON) metabolites, ochratoxin A (OTA) and aflatoxin M1 (AFM1), clearly indicating that the occupational environment contributes to internal exposure [13]. A study investigating occupational and dietary exposure of feed-handling workers to several mycotoxins in animal-producing farms and feed factories in Brazil reports the existence of exposure to fumonisins (FBs) in these work environments, considering their high concentrations in all airborne dust samples and the high exposure reflected in urinary biomarkers of workers [14].

In Italy, research on occupational exposure to mycotoxins has so far mainly focused on environmental monitoring and risk characterization. Italian studies and reviews highlighted the occurrence of airborne mycotoxins in livestock environments and identified animal care and feed-handling tasks as critical exposure scenarios [7]. Furthermore, recent Italian investigations on pig feed contamination have shown a widespread presence of toxigenic fungi and multiple mycotoxins, suggesting that feedstuffs may act as an important source of environmental release and consequently of workers’ exposure [15].

Despite these contributions, original data on exposure to mycotoxins in occupationally exposed populations are still extremely limited, and, to date, no Italian studies have specifically addressed urinary mycotoxin biomarkers in swine farmers. This work aims to contribute to the limited national evidence available, and to support a more comprehensive evaluation of exposure and risk in the livestock sector.

**Figure 1:**
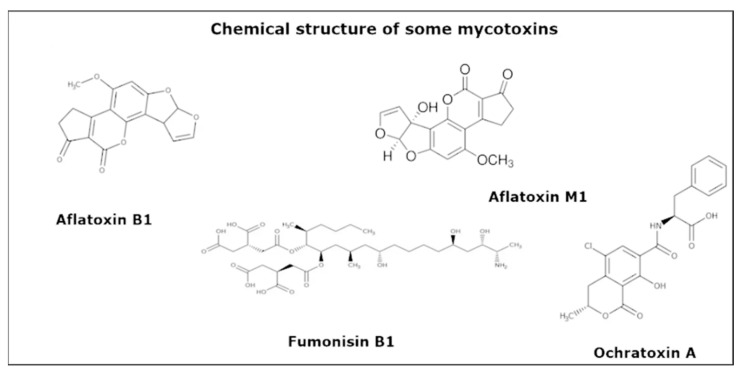
Chemical structures of AFB1, AFM1, FB1 and OTA.

Specifically, a new HPLC-MS/MS analytical method was developed in our laboratory and applied to measure urinary biomarkers of multiple mycotoxins (aflatoxin B1-AFB1, ochratoxin A-OTA, fumonisin B1-FB1) and aflatoxin M1-AFM1 (the hydroxylated metabolite of AFB1). Figure 1 shows the chemical structure of the four mycotoxins studied.

Five biomarkers of oxidative stress (8-oxoGua, 8-oxoGuo, 8-oxodGuo, 3NO_2_Tyr, 5MeCyt) were also measured to evaluate the effects of exposure on DNA, RNA and proteins. Correlating biomarkers of internal dose with biomarkers of biological effect represents a valuable approach for improving exposure assessment, prevention strategies, and health surveillance in occupational settings, providing a more integrated picture of workers’ internal burden and potential early health impact than exposure markers alone [8,13]. Moreover, the analysis of biomarkers of oxidative stress represents an added value, as it may allow the evaluation of early effects of mycotoxin-induced oxidative stress.

## 2. Materials and Methods

### 2.1. Chemicals and Reagents

Analytical reference standards for aflatoxin B1(AFB1), fumonisin B1(FB1), aflatoxin M1 (AFM), ochratoxin A (OTA), 5-methylcytidine (5-MeCyt) ≥ 99.9%, cotinine d_3_ solution and glacial acetic acid, 30% ammonia solution, dimethyl sulfoxide, sodium hydroxide solution (50–52% in water), CHROMASOLV^®^ gradient-grade methanol (≥99.9%), acetonitrile for HPLC/MS (≥99.9%), and low-benzene carbon disulfide were purchased from Sigma-Aldrich (Saint Louis, MO, USA). 8-oxy-7,8-dihydroguanine (8-oxoGua), 8-oxy-7,8-dihydro-2′-deoxyguanosine (8-oxodGuo) and 8-oxy-7,8-dihydroguanosine (8-oxoGuo) were obtained from Spectra 2000 srl (Rome, Italy). The isotope-labeled internal standards, (^13^C^15^N_2_)8-oxodGuo and (^13^C^15^N_2_)8-oxoGuo, were supplied by CDN Isotopes Inc. (Pointe-Claire, QC, Canada); (^13^C^15^N)8-oxoGua (purity 98%) was supplied by Cambridge Isotope Laboratories Inc. (Tewksbury, MA, USA). The 3-nitrotyrosine (3-NO_2_Tyr) was purchased from Cayman Chemical Company (Ann Arbor, MI, USA) and 3-NO_2_Tyr-d_3_ from TRC (Toronto, ON, Canada). Sep-Pak, C18, 6 cc cartridges were obtained (Waters S.p.A, Milan, Italy). Ultrapure water was produced using a Milli-Q Plus purification system (Millipore, Milford, MA, USA). Anotop 10LC syringe filters (0.2 µm pore size, 10 mm diameter) were obtained from Whatman Inc. (Maidstone, UK). A Synergi Luna C8 column (250 mm × 4.6 mm) and Kinetex Polar C18 column (100 Å, 150 mm × 4.6 mm, 2.6 µm) were supplied by Phenomenex (Torrance, CA, USA). The Analyst software version 7.1 (2024) has been used for instrumental data elaboration.

### 2.2. Subjects, Sample Collection and Study Design

Workers included in the study were recruited from 20 swine farms located in four provinces of the Calabria region (15,082 km^2^):

- Catanzaro (CZ; 7 farms);
- Reggio Calabria (RC; 5 farms);
- Cosenza (CS; 5 farms);
- Crotone (KR; 3 farms).

Farms were selected by both geographic distribution and convenience, mainly based on the willingness of farmers to participate in the survey. Inclusion criteria for the exposed group comprised individuals involved in farming activities, including direct or indirect contact with animals, feed, or environmental matrices. Participants were required to have a minimum duration of employment [e.g., ≥6 months] to ensure consistent exposure. Exclusion criteria included the presence of chronic diseases, ongoing pharmacological treatments, or other conditions potentially affecting biomarker levels, as well as recent changes to occupational status.

The control group consisted of individuals involved in administrative tasks and not occupationally exposed to swine farming or related agricultural activities. The farms belonged to intensive (9/20) and non-intensive (11/20) types of breeding. Intensive-type farms were considered those in which animals were in crowded conditions; swine holdings with non-intensive breeding systems were those in which animals were not confined between fences.

Spot urine samples were collected in sterile plastic containers, divided into three aliquots in polypropylene screw-cap tubes and stored at −20 °C until analysis. One aliquot was used for the determination of mycotoxins, one for the creatinine concentration and the third one for the determination of urinary 8-oxoGua, 8-oxodGuo, 8-oxoGuo, 3-NO_2_Tyr and 5-MeCyt. All results were normalized to creatinine and expressed in µg/g creatinine. Urinary creatinine was determined by the method of Jaffè using the alkaline picrate test with UV/Vis detection at 490 nm [16].

Each sample was coded according to the following nomenclature: code name HU (Human Urine) followed by a sequential number and 2-letter prefix indicating the Italian province (i.e., HU01KR, HU02CS, etc.).

The study was approved by the Ethics Committee of the “G. Martino” University Hospital in Messina, Italy (prot. 02-24; November 13 2023); participation was voluntary and the workers provided their informed consent to participate in the study; personal (e.g., sex, age, smoking and dietary habits, etc.), work (e.g., work hours per day/week, time of employment), and health-related data—with particular attention to respiratory symptoms, as well as gastrointestinal and skin symptoms—were collected via questionnaires. All data collected were based on self-reporting.

### 2.3. Sample Preparation for Mycotoxin Analysis

Urine sample (5 mL) were slightly acidified with 1 mL of 2% (*v*/*v*) acetic acid in water, spiked with 50 µL of a 10 mg/L cotinine d_3_ solution, and purified with solid-phase extraction (SPE). Sep-Pak cartridges were conditioned with 3 mL methanol, followed by 3 mL of 2% acetic acid in water. After sample loading, (5 mL urine sample) cartridges were washed with 3 mL of 2% *v*/*v* acetic acid in water. Elution was performed with 2 mL of methanol. Eluates were filtered through 0.2 µm Anotop filters and injected into the HPLC system for analysis.

Data acquisition and processing were performed using Analyst 7.1 software.

### 2.4. Method Validation

The method was validated using calibration standards and quality control (QC) samples. Linearity was assessed by evaluating the calibration range and the coefficient of determination (R^2^) of the regression model. The limit of detection (LOD) and the lower limit of quantification (LLOQ) were calculated as (3.3σ)/S′ and (10σ)/S’, respectively, where σ represents the standard deviation and S′ the slope of the calibration curve [17].

Accuracy and precision were determined by analyzing replicate QC samples at three concentration levels—low (2 μg/L), medium (5 μg/L), and high (20 μg/L)—for AFB1, OTA and FB1; and low (1 μg/L), medium (2 μg/L), and high (5 μg/L) for the AFM1 metabolite. The matrix effect was evaluated using the following formula:

Matrix effect (%) = (slope of the calibration curve obtained from spiked synthetic urine/slope of the standard calibration curve) × 100%.

#### 2.4.1. Calibration Curve and Quality Controls (QC) for Mycotoxins

Calibration curves (Figures S1–S4) were prepared using seven concentrations of AFB1, OTA, FB1 and AFM1, with a constant concentration of the internal standard (ISTD). All samples were spiked with cotinine d_3_ to obtain a final concentration of 100 µg/L.

The concentration levels were as follows: 0, 0.5, 1.0, 2.0, 10.0, 20.0 µg/L for AFB1, OTA, FB1 and 0, 0.25, 0.5, 1.0, 2.0, and 5.0 µg/L for AFM1. Calibration curves were prepared both in urine and in methanol. Similarly, the calibration curves of the five biomarkers of oxidative stress (8-oxoGua, 8-oxoGuo, 8-oxodGuo, 3NO_2_Tyr, 5MeCyt) are showed in Figure S5.

#### 2.4.2. HPLC-MS/MS Conditions for Mycotoxins

An AB Sciex API4000 HPLC/MS/MS system (SCIEX Headquarters AB Sciex LLC 250 Forest Street Marlborough, MA 01752, USA.) was used, equipped with a Synergi Luna C8 column (250 × 4.6 mm). A gradient of acetonitrile and acetic acid at 1% (*v*/*v*) in water was employed as the mobile phase used. The triple-quadrupole mass spectrometry detector was operated in positive ion mode, using a Turbo Ion-Spray source (SCIEX Headquarters AB Sciex LLC 250 Forest Street Marlborough, MA 01752, USA). The following ion transitions were monitored: *m*/*z* 313 → 241 for AFB1, *m*/*z* 722→334 for FB, *m*/*z* 404 → 358 for OTA, and *m*/*z* 180.3 → 80.1 for cotinine d_3_. The column temperature was maintained at 40 °C, the mobile phase flow rate was 1 mL/min, and the injection volume was 10 µL. The retention time was 8.9, 7.0, 5.8 and 12.3 min for AFB1, AFM1, FB1 and OTA respectively. The total run time was 15 min.

**Figure 2:**
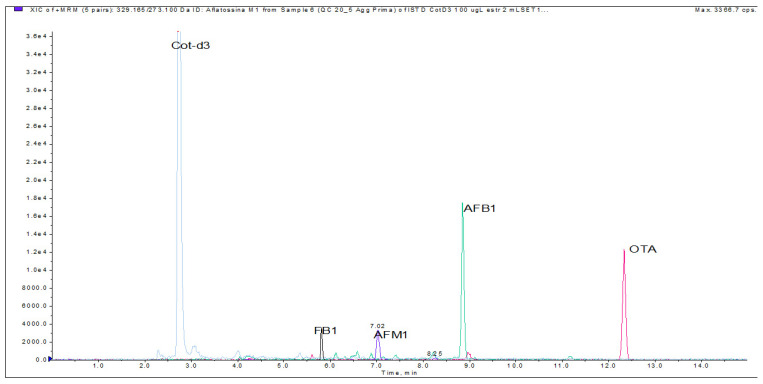
Chromatogram of a urine sample.

Figure 2 shows the chromatogram of a urine sample, enriched with pure mycotoxin standards, where peaks of all the mycotosins are clearly showed, and fully separated from interferents.

### 2.5. Biomarkers of Oxidative Stress Analysis

The determination of biomarkers of oxidative stress was conducted in accordance with the method reported by Andreoli et al. [18], with some methodological adjustments concerning the sample thawing process, the selection of dilution solvents, the chromatographic column, and the composition of the mobile phases. Samples were thawed in water at 37 °C, centrifuged, and filtered. The obtained supernatant was spiked with the internal standard mixture and subsequently injected into the HPLC–MS/MS system for analysis. Detection was achieved in the positive electrospray ionization mode. Chromatographic separation was carried out on a Kinetex Polar C18 column (100 Å, 150 × 4.6 mm, 2.6 µm).

Urine sample analyses were performed using a Series 200 LC quaternary pump (PerkinElmer, Norwalk, CT, USA) coupled to an AB/Sciex API 4000 triple-quadrupole mass spectrometer equipped with a Turbo Ion-Spray (TIS) interface.

## 3. Results and Discussion

### 3.1. Validation of the Analytical Method

Accuracy was assessed by analyzing a set of blank urine samples fortified with appropriate amounts of pure standards to obtain exact nominal concentrations. Accuracy was expressed as the mean percentage ratio between the measured concentration (µg/L) and the nominal concentration for each level. Measurements were repeated five times on the same day (intra-day accuracy) and one on each of the following two non-consecutive days (inter-day accuracy). Precision was evaluated as the repeatability of the analytical result and expressed as the mean coefficient of variation (CV%) from five independent sets of analyses performed on the same day (intra-day precision), and across three different days (inter-day precision).

For AFB1, intra-day accuracy ranged from 83% to 91%, for OTA from 107% to 133%, for FB1 from 85% to 94%, and for AFM1 from 129% to 139%. The corresponding CV% values were 9.8%, 5.8%, 6.2% and 8.3% respectively.

Inter-day accuracy was evaluated by performing analyses over three consecutive days. It ranged from 86% to 93% for AFB1, from 110% to 133% for OTA, from 94% to 101% for FB1, and from 108% to 150% for AFM1. The corresponding CV% values were 13%, 6%, 23% and 11% respectively. Calibration curves were constructed using seven levels, ranging from 0 to 20 µg/L for AFB1, OTA, and FB1; and from 0.0 to 5 µg/L for AFM1 in urine. Linearity was confirmed with a regression coefficient (R^2^) greater than 0.998 for all the biomarkers.

The mean solid-phase extraction (SPE) recovery was 100% for AFM1, 89% for OTA, 136% for AFB1, and 87% for FB1.

The matrix effect, evaluated based on the internal standard (ISTD) analytical response, was approximately 80%. The matrix effect refers to the change in the instrumental response caused by the sample matrix (e.g., urine compared to methanol) and is mainly due to co-eluting compounds that can suppress or, in some cases, enhance ionization in the mass spectrometry source. This issue can be mitigated through the use of isotopic dilution; however, a reduction in sensitivity is generally unavoidable. The method shows very clean chromatographic peaks characterized by low background noise and minimal interference. Quantification was easy and rapid.

Regarding the sensitivity of the analytical method, Table 1 presents the limits of detection (LODs) and limits of quantification (LOQs) achieved in the biological monitoring analyses, along with a comparison with those reported in recent comparable studies.

The comparison between the LOD and LOQ obtained in the present study and those reported in the literature highlights the overall good analytical performance of the applied method, which is fully consistent with—and in several cases superior to—most previously published studies on urinary biomonitoring of mycotoxins.

For AFB1, the LOD achieved in this study (0.07 μg/L) is markedly lower than the value reported by Escrivá L. et al. [19], who reported an LOD of 0.4 μg/L, and is comparable to or better than the value (0.1 μg/L) measured by Chen M. et al. [23]. The LOQ (0.2 μg/L) is also lower than those reported in earlier studies and falls within the same order of magnitude as more recent investigations, indicating improved analytical sensitivity for an analyte that is notoriously challenging to quantify in urine matrices.

For AFM1, the method achieved an LOD of 0.02 μg/L which is substantially lower than that reported in a study conducted on swine workers (LOD 0.11 μg/L) [13] and comparable to the lowest values described in the most recent study [25]. The LOQ (0.06 μg/L) is likewise competitive and enables reliable quantification at low concentration levels, which is particularly relevant for occupational exposure scenarios characterized by intermittent and variable exposure patterns.

With regard to FB1, the LOD and LOQ obtained (0.05 and 0.2 μg/L, respectively) are appropriate for human biomonitoring studies, even though some very recent publications based on ultra-high-sensitivity instrumentation [21,25] report lower values. However, it should be noted that such studies are often conducted under controlled experimental conditions or in general population samples, whereas the present method was applied to real-world samples from occupationally exposed workers, thereby balancing analytical sensitivity with robustness and reproducibility.

For OTA, the LOD obtained in this study (0.09 μg/L) is substantially lower than those reported in several earlier studies, including Pallarés N. et al. [22] and Escrivá L. et al. [19], which reported limits in the unit range. Although the method does not reach the extremely low detection levels achieved in some recent methodological studies [21,25], it nevertheless allows reliable quantification of OTA at concentrations of toxicological and occupational relevance, as reflected by the LOQ of 0.3 μg/L.

Overall, our findings indicate that the analytical method developed in the present study shows adequate and competitive sensitivity compared with the existing literature. It is particularly well suited for biomonitoring studies in occupational settings. The combination of satisfactory detection limits, applicability to multiple mycotoxins, and successful application to urine samples from pig farmers further supports the robustness of the method and the comparability of the generated data with major European and international studies.

### 3.2. Mycotoxin Biomarker Occurrence in Human Urine

A total of 65 spot urine samples were collected, including 35 samples from workers involved in animal management and care, and 30 samples from personnel working in offices without expected occupational exposure to mycotoxins as the “control group”.

Table 2 presents the occurrence of mycotoxin biomarkers in urine samples collected from both workers and control subjects, reported both as an absolute number and as a percentage of positive samples. For each analyzed mycotoxin and/or metabolite, mean concentrations together with the corresponding standard deviations are also provided.

Samples with levels of mycotoxins and/or their metabolite greater than the respective limit of detection (LOD) were considered positive.

The results indicate a low but measurable internal exposure to multiple mycotoxins in the study population, with differences between exposed farmers and the control group.

Among the mycotoxins investigated, AFM1 emerged as the most frequently detected biomarker in the exposed group; its concentrations were above the LOD in eight subjects (22.8%) and four samples (11.4%) exceeded the LOQ, whereas only 10% of control samples were above the LOD and none exceeded the LOQ. These findings suggest a possible occupational contribution to internal AFM1 exposure, in line with previous studies carried out in similar settings, which have reported associations between feed handling, animal husbandry activities, and potential exposure scenarios [7].

AFB1 was detected sporadically and exclusively in the exposed group. Only 5.7% of workers showed concentrations above the LOD and one sample exceeded the LOQ, while it was not detected in any control sample.

Although the urinary concentrations of AFB1 detected in workers were low, their presence is of particular concern due to the well-established carcinogenicity of this compound. AFB1 is classified by the International Agency for Research on Cancer (IARC) as a Group 1 human carcinogen, with sufficient evidence linking exposure to the development of hepatocellular carcinoma through genotoxic mechanisms involving DNA adduct formation. In addition, for genotoxic carcinogens such as AFB1, no safe exposure threshold can be assumed, and even low-level, chronic exposure may contribute to cancer risk. Therefore, the detection of AFB1 biomarkers in urine, even at low concentrations, provides relevant evidence of internal exposure and highlights the need to consider occupational environments as potential contributors to cumulative lifetime exposure and associated long-term health effects [5,26,27].

OTA was detected above the LOD in both groups, with a higher frequency in controls (43.3%) compared with exposed workers (31.4%), while no samples exceeded the LOQ in either group. Mean OTA concentrations observed in this study were comparable between exposed workers and control subjects (0.04 µg/L), suggesting that background exposure, most likely of dietary origin, represents the main contributor to body burden. This finding is consistent with the extensive literature identifying OTA as a ubiquitous food contaminant and demonstrating its widespread presence in the general population [28,29,30].

In the present study, FB1 was not detected in exposed workers, whereas concentrations above the LOD (0.10 µg/L) were observed in a small proportion of control subjects (2/30). Overall, mean FB1 levels were extremely low, suggesting minimal systemic exposure to this metabolite. This finding is noteworthy given that environmental and feed monitoring studies consistently report widespread occurrence of fumonisins, particularly in maize-based products [31].

The apparent discrepancy between environmental levels and internal exposure may be explained by the low oral bioavailability of FB1 in humans and its rapid elimination, which limits its detectability in biological matrices and may result in low prevalence in biomonitoring despite its widespread presence in the environment [32,33,34].

Regarding co-exposure to different mycotoxins, seven workers showed co-exposure to AFM1 and OTA and two workers showed co-exposure to AFB1, AFM1 and OTA. Among controls, only two subjects presented simultaneous exposure to AFB1 and OTA.

Overall, mean urinary concentrations for all analytes were low and close to the analytical detection limits, which is consistent with intermittent and low-dose exposure patterns typical of occupational settings, rather than continuous high-level exposure. Nevertheless, the higher detection frequencies of AFM1 and AFB1 in exposed workers compared with controls support the hypothesis that pig farming activities can contribute to additional aflatoxin exposure beyond dietary sources.

These findings confirm the usefulness of urinary biomonitoring for capturing real-world occupational exposure to mycotoxins and provide original evidence from Italian pig farmers, a population for which human biomonitoring data were previously lacking.

### 3.3. Comparison with Earlier Environmental Monitoring Studies Conducted in the Same Italian Facilities

To further explore and interpret the study findings, mean values of mycotoxins/metabolite concentrations were stratified by individual farm, and additional information obtained from questionnaires—including the number of workers, type of breeding system, sex, smoking habits, and duration of employment—was incorporated into the analysis (Table 3). Both groups (workers and controls) have similar diets; consequently, it was hypothesized that the main difference in exposure to mycotoxins was work activities. Table 3 also reports the results of oxidative stress biomarkers.

Overall, the biomonitoring findings were in agreement with previously published results from environmental monitoring studies—including air, settled dust, and feed—carried out in the same swine farms by some of the authors of the present study [15].

As shown in Table 3, the highest mean urinary concentrations of AFB1 and AFM1 were observed in farm 02CS, where environmental investigations documented a relevant source of exposure. In this farm, feed samples were contaminated with four strains of *Aspergillus flavus*, three of which possessed all five key genes involved in the aflatoxin biosynthetic pathway; their aflatoxin-producing capability was confirmed by HPLC–MS/MS analyses. Moreover, aflatoxigenic *A. flavus* strains were also detected in bioaerosol and surface samples [unpublished data]. These findings underline the value of correlating human biomonitoring data with environmental contamination patterns to strengthen exposure assessment and to support a causal interpretation of occupational mycotoxin exposure.

Mycotoxin biomarkers remain overall low in both intensive and non-intensive settings; however, higher peak concentrations (notably for AFB1 and AFM1) are more frequently detected in intensive farms. In contrast, oxidative stress biomarkers display a clearer pattern, with 8-oxoGua levels generally elevated in intensive systems compared to non-intensive farms, where values are more heterogeneous. Similarly, 3-nitrotyrosine (3-NO_2_Tyr) shows increased levels in selected intensive settings, suggesting enhanced nitrosative stress.

Nevertheless, this trend is not fully consistent, as some non-intensive farms also present elevated oxidative stress markers, indicating that exposure is not solely driven by the farming system. This further confirms the nonspecific nature of these biomarkers of effect. These findings support a multifactorial exposure scenario, potentially influenced by environmental conditions, task-specific activities, and feed contamination.

Importantly, no clear association is observed between employment duration and biomarker levels. Both short- and long-term workers exhibit variable biomarker profiles, suggesting that recent exposure, working practices, and individual susceptibility may play a more relevant role than cumulative occupational history.

No statistically significant differences were observed in oxidative stress biomarkers between the exposed group and the controls. Table 3 shows between-farm variability in biomarkers of oxidative and nitrosative stress. Among all sites, farm 02CS presents slightly higher values of 8-oxoGua (46.29), 8-oxodGuo (7.40) and 3-NO_2_Tyr (11.10–12.58) than the other farms. This farm-specific pattern may reflect specific local conditions contributing to early biological effects or simply to a slightly higher number of individuals. In any case, these findings should be interpreted cautiously, as they do not allow causal attribution to a single exposure factor.

## 4. Conclusions

This paper provides original human biomonitoring data on mycotoxin exposure in Italian pig farmers, addressing a relevant knowledge gap at both national and European levels. By applying a multi-mycotoxin analytical approach to urine samples, the results confirm that pig farming activities may contribute to a measurable internal exposure to selected mycotoxins beyond background levels.

Although urinary concentrations were generally low and close to the analytical detection limits, differences between exposed workers and controls were observed for aflatoxin-related biomarkers, particularly AFM1 and, to a lesser extent, AFB1. These findings suggest that occupational tasks typical of livestock farming, such as feed handling and animal care, may represent episodic sources of exposure, consistent with the intermittent nature of mycotoxin release in agricultural environments. In contrast, the widespread detection of OTA in both groups supports the role of dietary exposure as a major determinant of background body burden, highlighting the complexity of disentangling occupational and non-occupational sources.

Furthermore, the study demonstrates the feasibility and usefulness of urinary biomonitoring for assessing real-world occupational exposure to mycotoxins. Even in scenarios characterized by low-dose and variable exposure, this approach proved capable of capturing internal doses that might be underestimated by environmental measurements alone. This reinforces the value of integrating biomonitoring into occupational risk assessment strategies for biological contaminants such as mycotoxins.

Future studies combining longitudinal sampling, larger populations and integrated environmental and biological monitoring are warranted to better characterize exposure dynamics, identify critical tasks, and support the development of targeted preventive measures. Within a One Health framework, improving the understanding of occupational mycotoxin exposure may ultimately benefit not only worker health, but also animal health and food safety.

## Figures and Tables

**Table 1:** Limits of detection (LODs) and limits of quantification (LOQs) of the analytical method and comparison with other papers.

Analyte	LOD *	LOQ *	Analytical Method	References
AFB1	7.0 × 10^−2^	0.20		This paper
AFM1	2.0 × 10^−2^	6.0 × 10^−2^	HPLC/MS/MS	
FB1	5.0 × 10^−2^	0.20		
OTA	9.0 × 10^−2^	0.3		
AFB1	0.4	1.20		Escrivá L. et al., 2017 [19]
OTA	8	20.00	LC/MS/MS	
AFM1	0.11	0.38		Viegas S. et al., 2019 [13]
OTA	1.1 × 10^−2^	0.04	LC/MS/MS	
AFB1	--	0.50		Tkaczyk A. et al., 2021 [20]
AFM1	--	0.50	LC/MS/MS	
OTA	--	1.50		
AFM1	7.0 × 10^−3^	0.25		Schmidt J. et al., 2021 [21]
OTA	4.0 × 10^−3^	1.0 × 10^−2^	UPLC/MS/MS	
FB1	4.0 × 10^−3^	1.0 × 10^−2^		
OTA	3.00	10.00	LC-ESI-QTOF-MS	Pallarés N. et al., 2022 [22]
AFB1	0.10	0.20		Chen M. et al., 2023 [23]
AFM1	5.0 × 10^−2^	0.10	LC/MS/MS	
OTA	0.20	0.40		
OTA	5.0 × 10^−2^	--	LC/MS/MS	McKeon H.P. et al., 2024 [24]
AFM1	1.0 × 10^−2^	6.0 × 10^−2^		Kuhn M. et al., 2025 [25]
FB1	1.0 × 10^−3^	2.0 × 10^−3^	LC/MS/MS	
OTA	2.0 × 10^−3^	8.0 × 10^−3^		

* Expressed in μg/L.

**Table 2:** Mycotoxin biomarkers detected in urine samples of workers and controls.

Groups		AFB1	AFM1	OTA	FB1
Exposed (*n* = 35)	Samples > LOQ	1 (2.8%)	4 (11.4%)	0	0
	Samples > LOD	2 (5.7%)	8 (22.8%)	11 (31.4%)	0
	Samples ≤ LOD	32 (91.4%)	23 (65.7%)	24 (68.6%)	35 (100%)
	Mean (Std) *	0.032 (±0.08)	0.022 (±0.04)	0.040 (±0.03)	0.014 (±0.01)
Controls (*n* = 30)	Samples > LOQ	0	0	0	0
	Samples > LOD	0	3 (10%)	13 (43.3%)	2 (6.7%)
	Samples ≤ LOD	30 (100%)	27 (90%)	17 (56.7%)	28 (93.3%)
	Mean (Std) *	0.012 (±0.02)	0.004 (±0.01)	0.042 (±0.04)	0.024 (±0.03)

* Expressed in μg/L.

**Table 3:** Mean urinary biomarker concentrations in workers, grouped by farm.

Farm ID	Type of Breeding	n. Workers	Males	Females	Smokers	Time of Employment (Years, Mean)	μg/g Creatinine						μg/g Creatinine		
							AFB1	AFM1	OTA	FB1	8-oxoGua	8-oxoGuo	8-oxodGuo	3-NO_2_Tyr	5-MeCyt
01KR	NI	3	3	0	2	2.5	0.02	0.00	0.02	0.01	29.17	4.67	5.57	2.16	4.95
03KR	NI	2	1	1	0	10	0.01	0.00	0.01	0.00	5.34	3.17	3.79	1.20	6.03
17KR	NI	1	1	0	0	16	0.08	0.02	0.03	0.00	5.49	5.64	5.69	2.52	10.65
02CS	I	4	3	1	2	13	0.14	0.07	0.06	0.02	46.29	4.29	7.40	11.10	12.58
04CS	NI	1	1	0	1	16	0.01	0.00	0.01	0.04	3.75	6.38	9.08	2.28	15.85
05CS	I	3	3	0	2	5	0.01	0.02	0.02	0.03	48.23	4.62	6.60	4.78	15.25
06CS	I	2	1	1	1	2.5	0.02	0.07	0.09	0.02	11.60	6.34	5.27	3.04	10.38
07CZ	NI	1	1	0	1	30	0.01	0.00	0.01	0.01	0.15	4.59	5.78	3.64	7.36
18CS	NI	1	1	0	0	12.5	0.03	0.00	0.04	0.02	NP	NP	NP	NP	NP
08CZ	I	1	1	0	0	16	0.02	0.02	0.03	0.02	38.67	5.58	2.67	1.16	5.58
12CZ	I	3	2	1	0	15	0.01	0.05	0.06	0.01	19.29	6.37	8.35	3.14	10.36
13CZ	I	2	1	1	0	16	0.02	0.00	0.05	0.00	1.55	7.74	4.42	1.72	9.50
14CZ	I	1	1	0	0	2.5	0.04	0.00	0.05	0.00	NP	NP	NP	NP	NP
15CZ	NI	2	2	0	0	12.5	0.01	0.00	0.03	0.00	5.24	5.66	3.96	1.91	6.46
16CZ	NI	1	1	0	0	12.5	0.02	0.02	0.11	0.00	8.29	6.21	5.42	2.35	10.15
09RC	I	2	2	0	1	16	0.01	0.01	0.02	0.03	27.81	7.05	8.53	5.85	18.82
10RC	I	1	1	0	0	13	0.01	0.01	0.02	0.02	40.51	2.46	1.60	0.44	9.25
11RC	NI	1	1	0	1	13	0.01	0.00	0.07	0.00	3.32	4.93	8.25	4.33	6.09
19RC	NI	1	1	0	1	2.5	0.01	0.01	0.03	0.00	3.01	3.82	4.90	3,60	33.06
20RC	NI	2	2	0	0	2.5	0.01	0.01	0.03	0.00	11.29	3.76	4.29	2.24	5.43

I: intensive type of breeding; NI: non-intensive type of breeding; NP: not performed due to insufficient sample.

## Data Availability

All data that support the findings of this study are available on reasonable request to the corresponding author.

## Supplementary Materials

The following supporting information can be downloaded at: https://www.mdpi.com/article/10.3390/toxics14070562/s1, Figure S1. Aflatoxin M1 calibration curve; Figure S2. Ocratoxin A calibration curve; Figure S3. Aflatoxin B1 calibration curve; Figure S4. Fumonisin B1 calibration curve; Figure S5. Calibration curves for each oxidative stress biomarker.

## Abbreviations

The following abbreviations are used in this manuscript:

AFB1	Aflatoxin B1
AFM1	Aflatoxin M1
OTA	Ochratoxin A
FB1	Fumonisin B1
IARC	International Agency for Research on Cancer
