# Supplementary material for: Biomonitoring of Occupational Exposure to Mycotoxins Among Swine Farm Workers: An Italian Pilot Study
Source: Toxics. 2026 Jun 27;14(7):562. doi: 10.3390/toxics14070562 (PMC13416590; doi:10.3390/toxics14070562)
Supplement: Supplementary file 1 [file toxics-14-00562-s001.zip › toxics-4357057-supplementary.pdf]

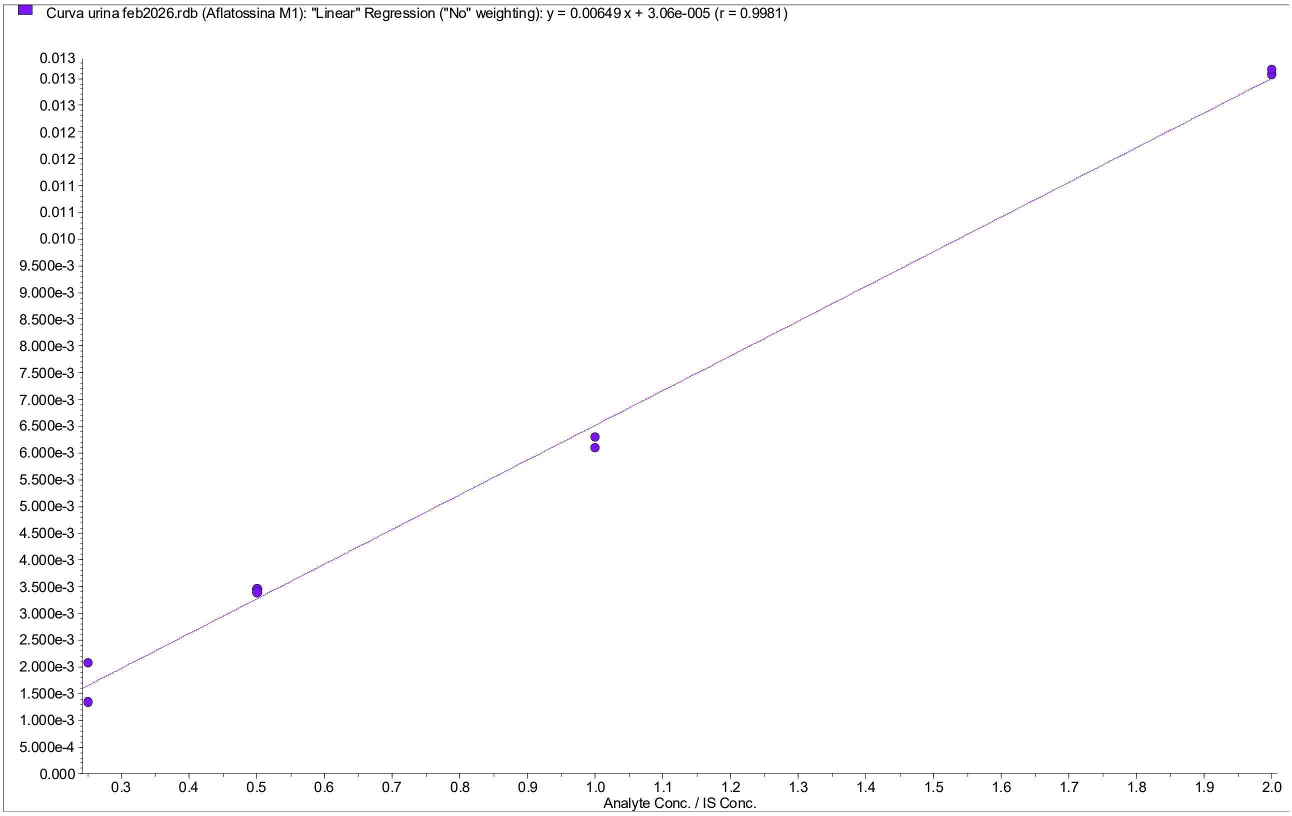


Figure S1. Aflatoxin M1 calibration curve

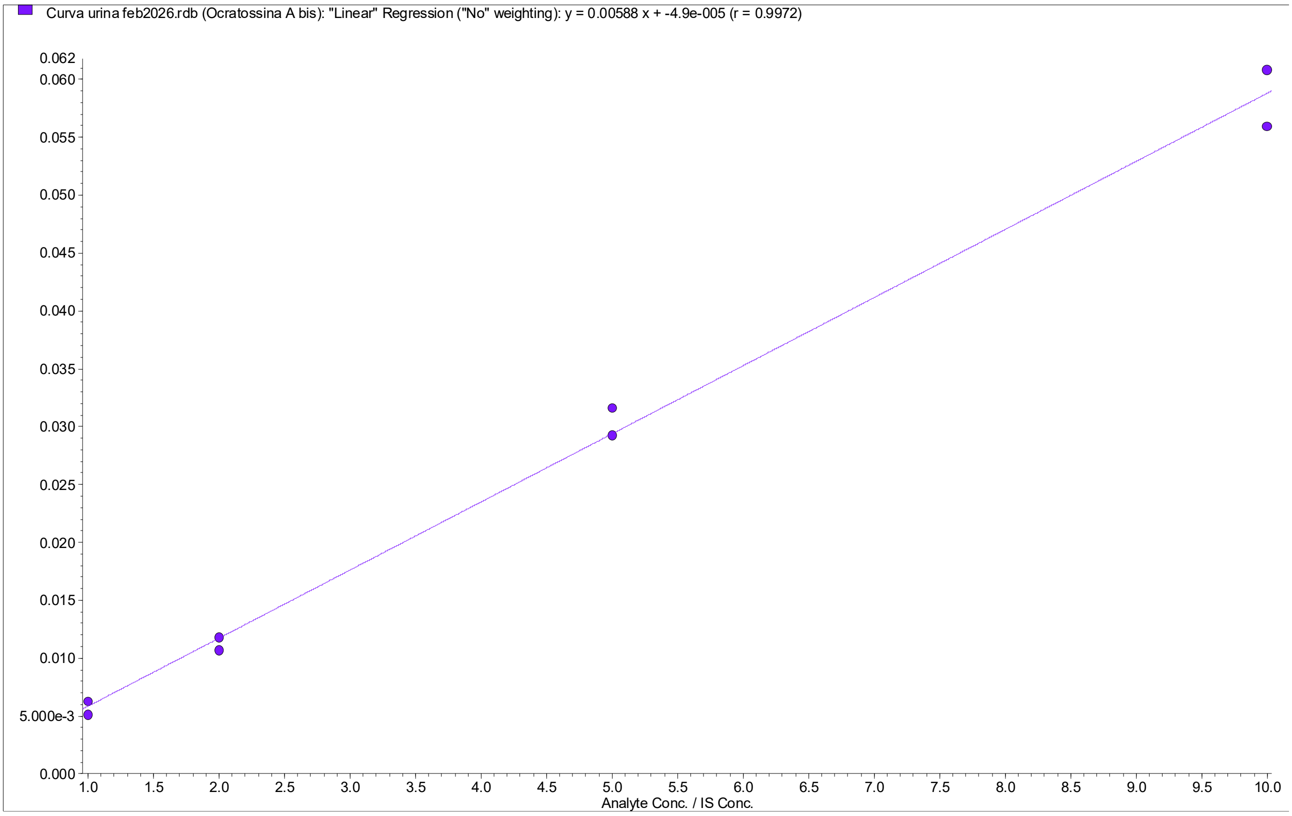


Figure S2. Ocratoxin A calibration curve

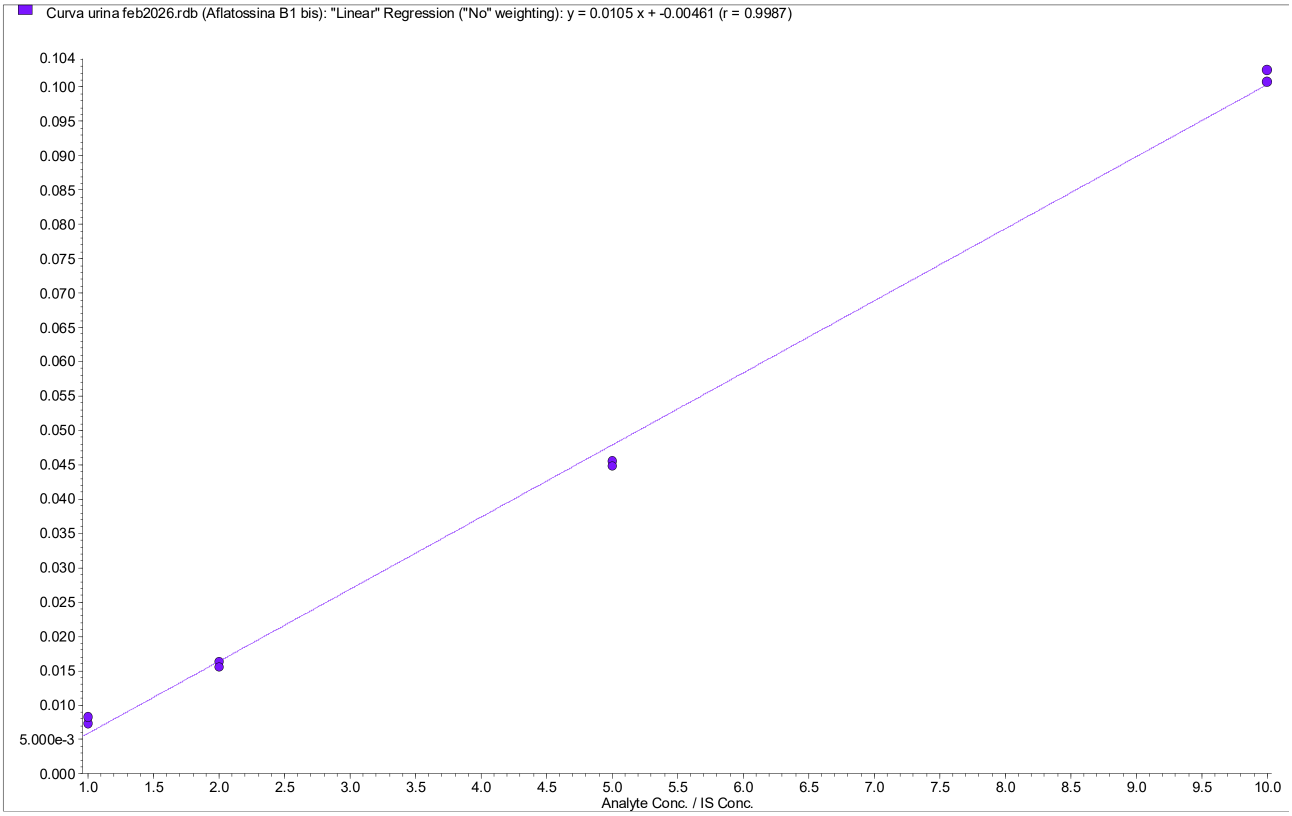


Figure S3. Aflatoxin B1 calibration curve

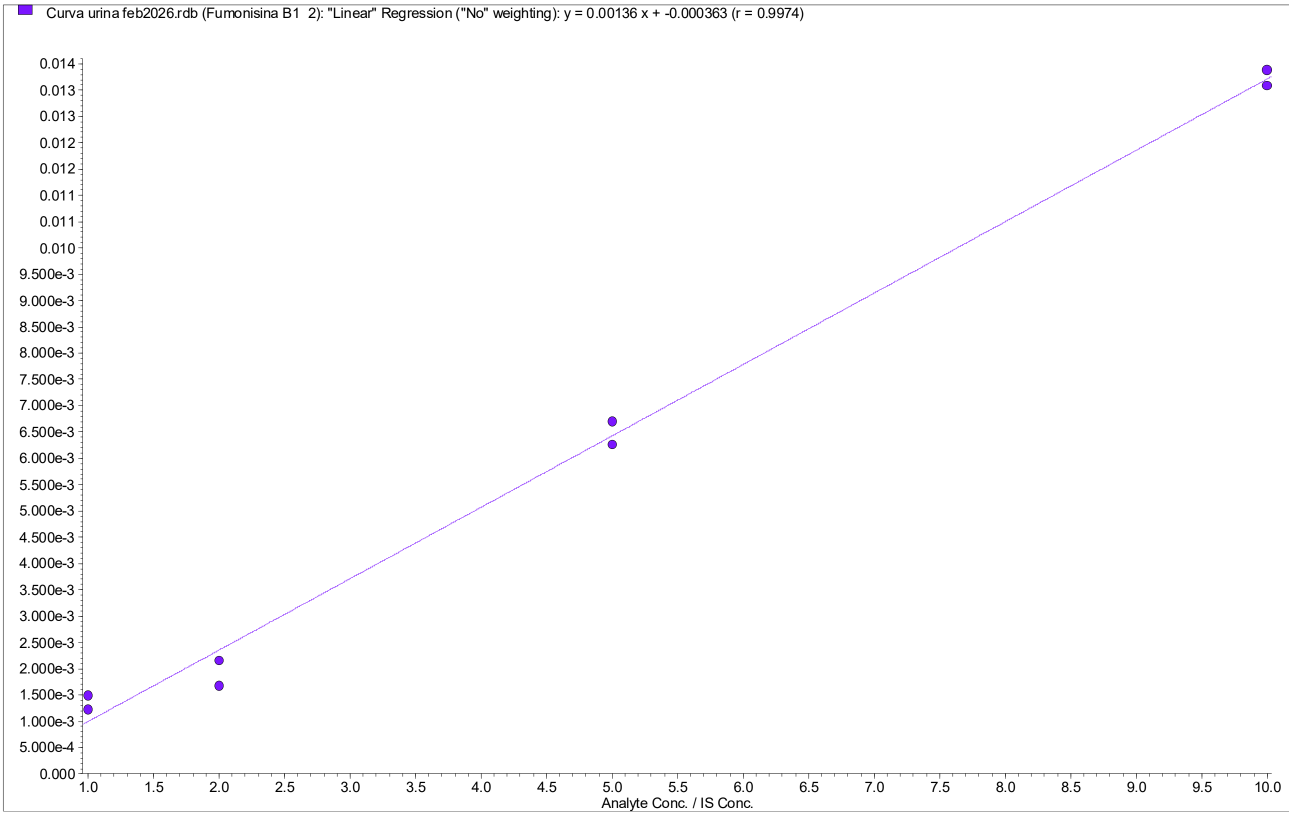


Figure S4. Fumonisin B1 calibration curve

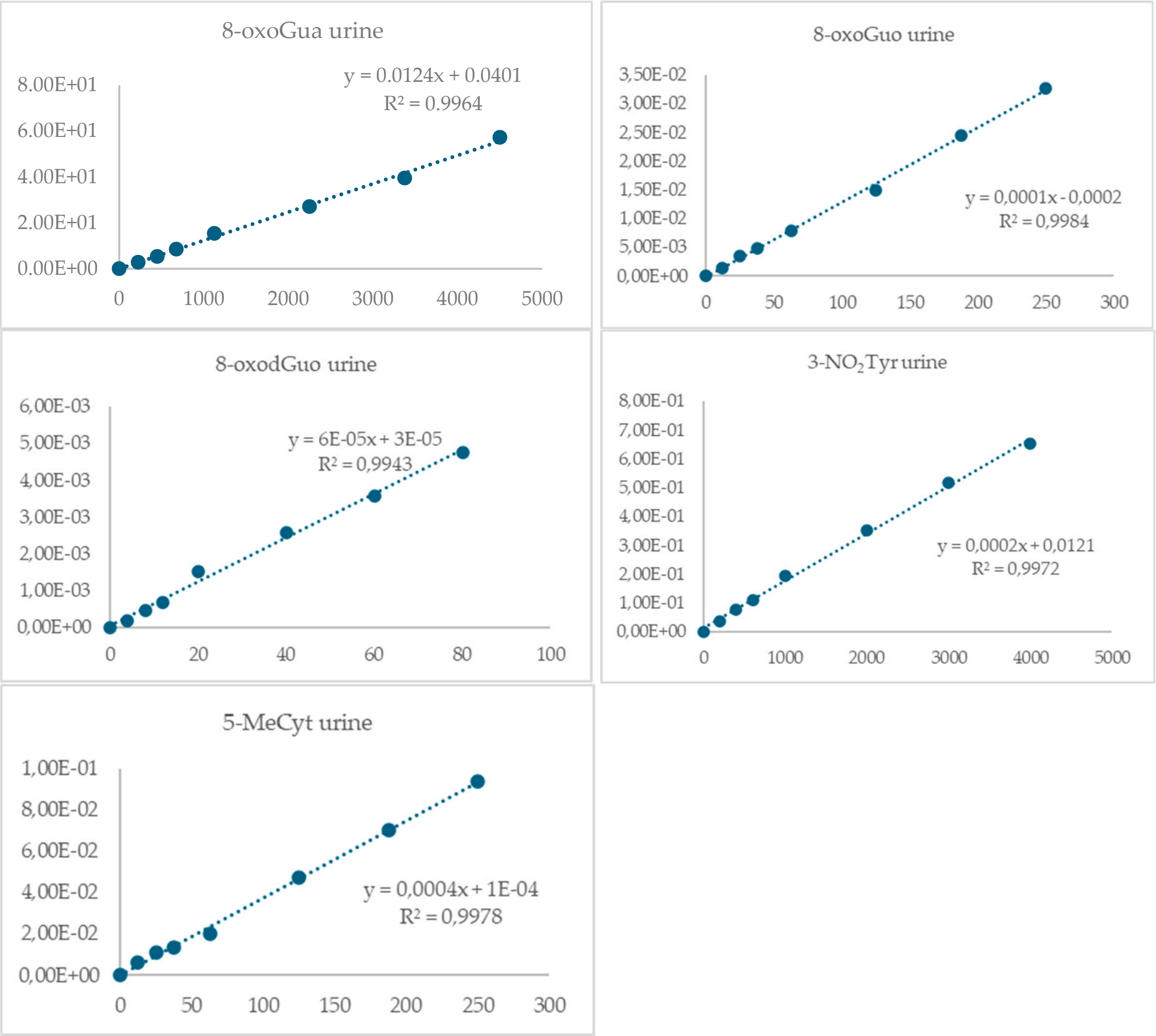


Figure S5. Calibration curves for each oxidative stress biomarker
